# Methylation changes in association with early life stress and trauma-focused psychotherapy in treatment-resistant depression

**DOI:** 10.1192/j.eurpsy.2023.313

**Published:** 2023-07-19

**Authors:** R. Carvalho Silva, C. Hohoff, P. Martini, M. Bortolomasi, R. Bazzanella, V. Menesello, M. Gennarelli, B. T. Baune, A. Minelli

**Affiliations:** 1 Department of Molecular and Translational Medicine, University of Brescia, Brescia, Italy; 2 Department of Psychiatry, University of Münster, Münster, Germany; 3 Psychiatric Hospital “Villa Santa Chiara”, Verona; 4 Genetics Unit, IRCCS Istituto Centro San Giovanni di Dio Fatebenefratelli, Brescia, Italy; 5 Department of Psychiatry, Melbourne Medical School, University of Melbourne, Melbourne; 6 The Florey Institute of Neuroscience and Mental Health, The University of Melbourne, Parkville, VIC, Australia

## Abstract

**Introduction:**

Early life stress (ELS) associates with unfavourable outcome in Major depressive disorder (MDD) and treatment-resistant depression (TRD). Trauma-focused psychotherapy benefits TRD patients exposed to ELS. Epigenetic processes are altered in stress-related disorders, but few studies show epigenetic signatures associated with trauma.

**Objectives:**

We performed an epigenome-wide association study (EWAS) to explore the relation between methylation changes in TRD patients characterized for recent and ELS and trauma-focused psychotherapy outcomes.

**Methods:**

Thirty TRD patients participated. They underwent psychotherapy, from which 12 cognitive behavioural therapy and 18 Eye Movement Desensitization and Reprocessing (EMDR). We used validated interviews and questionnaires for symptom evaluation and stress exposure. Patients were evaluated at T0 (baseline), T8 (end of psychotherapy), T12 (follow-up) and T26. Methylation was profiled with Illumina Infinium EPIC array for T0, T8 and T12. Methylation levels were quantified after quality control and normalization using ChAMP R package. We tested the association between B-values for each CpG site (each probe set) and each phenotype/condition using a linear model approach (with paired values) as implemented in the Limma R package. P-values were adjusted using Benjamini & Hochberg method. Probe sets were considered significant with an adjusted p-value q ≤ 0.05. CpG site annotation was performed using IlluminaHumanMethylationEPICanno.ilm10b2.hg19 R package (hg19 genome reference).

**Results:**

Association analyses between baseline methylation levels and emotional abuse resulted in two significant probe sets annotated in *SLCO4A1* (p=1,72E-08; q=0,008), involved in sodium independent transmembrane substrate transport, and *GPNMB* (p=1,53E-07; q=0,022), involved in cell differentiation. Associations between baseline methylation levels and physical abuse resulted in one significant probe set annotated in *DDIT4L* (p=4,77E-08; q=0,035), involved in cell growth.

In longitudinal analyses, association between T0-T8 methylation levels and response at T8 resulted in two significant probe sets annotated in *PLEKHB1* (p=3,54E-08; q=0,013), involved in cell differentiation, and *NUDT4P2* (p=1,34E-07; q=0,032). Longitudinal T12-T0 EWAS analyses in patients undergoing EMDR resulted in 44 significant probe sets annotated in genes, highlighting *MAD1L1* (p=6,28E-07; q=0,035), involved in cell division, and *TNFAIP3* (p=3,00E-06; q=0,045), which regulates immunity.

**Conclusions:**

We identified epigenetic signatures of ELS in TRD patients, suggesting that ELS may modulate the intensity of epigenetic alterations. Longitudinal methylation analyses along psychotherapy showed significant genes in relation to response, especially for patients undergoing EMDR. Some genes are associated with post-traumatic stress disorder (*MAD1L1*) and anxiety disorders and MDD (*TNFAIP3*).

**Disclosure of Interest:**

None Declared

